# Chinese EFL learners different from English natives in cataphora resolution: Evidence from eye-tracking studies

**DOI:** 10.3389/fpsyg.2023.1126673

**Published:** 2023-02-23

**Authors:** Tao Wang, Mingyao Geng, Yue Wang, Min Zhao, Tongquan Zhou, Yiming Yang

**Affiliations:** ^1^School of Psychology, Qufu Normal University, Qufu, China; ^2^School of Translation Studies, Qufu Normal University, Rizhao, China; ^3^College of Foreign Languages, Qufu Normal University, Qufu, China; ^4^School of Foreign Languages, University of Jinan, Jinan, China; ^5^School of Foreign Languages, Southeast University, Nanjing, China; ^6^School of Linguistic Sciences and Arts, Jiangsu Normal University, Xuzhou, China; ^7^Collaborative Innovation Center for Language Ability, Jiangsu Normal University, Xuzhou, China

**Keywords:** cataphora resolution, Chinese EFL learners, active search mechanism, L2 proficiency, eye-tracking

## Abstract

Previous studies on English natives have shown that encountering an English cataphoric pronoun triggers an active search for its antecedent and this searching process is modulated by syntactic constraints. It remains unknown whether the conclusion is universal to EFL (English as a Foreign Language) learners, particularly those with distinct L1 like Chinese in linguistic typology. Therefore, this study used two eye-tracking experiments to investigate how Chinese EFL learners resolve English cataphora. The experiments adopted the gender-mismatch paradigm. Experiment 1 investigated whether Chinese EFL learners with different proficiency would adopt the similar processing pattern to English natives and found that gender congruency elicited longer reading times than gender incongruency between the first potential antecedent and the cataphoric pronoun, the effect early observed in high-proficiency relative to low-proficiency learners. Experiment 2 explored whether the cataphora resolution process was modulated by Binding Principle B and revealed that longer first fixation durations and first pass reading times were observed in gender-mismatch than in gender-match conditions no matter the antecedents are binding-accessible or not while longer regression path durations occurred in gender-mismatch than in gender-match conditions only as the antecedents are binding-accessible. Taken together, these results indicate that Chinese EFL learners also adopt an active search mechanism to resolve cataphoric pronouns, yet along a processing path distinct from English natives’. Specifically, Chinese EFL learners predictively link a cataphoric pronoun to the first potential antecedent in the sentence but only a gender-matching antecedent can prompt them to engage in deep processing of the antecedent. Moreover, the processing time varies with the learners’ English proficiency. Furthermore, unlike native English speakers’ early application of syntactic constraints in their cataphora resolution, Chinese EFL learners try to establish co-reference relations between cataphoric pronouns and antecedents regardless of following or flouting Binding Principle B in early processing stages whereas they exclusively link the cataphoric pronouns to the binding-accessible antecedents in late processing stages. This study adds evidence to the Shallow Structure Hypothesis whereby L2 learners resort to lexical prior to syntactic cues to process sentences in general, which is just opposite to the fashion adopted by the natives.

## 1. Introduction

Cohesive devices abound in human language. Of all the cohesive devices, anaphora is used in English more often than not. Anaphora refers to the linguistic phenomenon whereby the interpretation of one linguistic element (anaphor) depends on the interpretation of another (antecedent) (Huang, 2000; Kazanina, 2005). Based on the linear position between the two linguistic elements, anaphora is classified into two types: forwards anaphora where the forwards anaphor appears after its antecedent and backwards anaphora (also referred to as cataphora) where the backwards anaphor (cataphor) appears before its antecedent. The process of finding the antecedent for an anaphor is called anaphora resolution (Mitkov, 2002). Anaphora resolution has been a central topic in the field of psycholinguistics for the last few decades (e.g., Felser et al., 2009; Felser and Cunnings, 2012; Chow et al., 2014; Cunnings et al., 2014; Wu, 2016, 2017; Cunnings et al., 2017; Yu and Zhai, 2017; Liang et al., 2018; Yu and Dong, 2019; Liu, 2020; Tang and Wen, 2020), for the correct interpretation of anaphors underlies successful sentence comprehension. Though both being the commonly used cohesive devices in English, backwards anaphora has received much less attention than forwards anaphora in empirical studies. To date, the majority of cataphora resolution studies have been conducted on native speakers (Kazanina et al., 2007; Kazanina and Phillips, 2010; Clackson and Clahsen, 2011; Yoshida et al., 2014; Pablos et al., 2015; Patterson and Felser, 2019; Kush and Dillon, 2021) while few on L2 learners (Rodríguez, 2008; Bertenshaw, 2009; Drummer and Felser, 2018; Wu et al., 2019). Although the EFL learners in the L2 cataphora resolution studies exhibited similar processing patterns as English natives, they basically have a L1 similar to English in linguistic typology. As a consequence, it remains unknown how English learners with a typologically distinct L1 resolve English cataphora. Since Chinese as a semantics-driven language contrasts with English as a syntax-driven language, an examination is required to test whether this typological difference influences Chinese EFL learners’ online processing of English cataphoric pronouns. Against this background, the current study aims to investigate Chinese EFL learners’ online processing of English cataphoric pronouns.

Previous cataphora resolution studies on native speakers have shown that encountering a cataphoric pronoun triggers an active search for its antecedent (Cowart and Cairns, 1987; van Gompel and Liversedge, 2003; Filik and Sanford, 2008). In van Gompel and Liversedge’s (2003) eye-tracking study, the gender-mismatch paradigm was adopted for the first time to test whether the use of morphological information preceded the computation of coreference relations during pronoun resolution. They used materials like those in (1), in which the gender of the first noun phrase in the main clause was manipulated to either match or mismatch the cataphoric pronoun in the subordinate clause.

(1) a. gender-match

When he was at the party, *the boy* cruelly teased the girl during the party games.

b. gender-mismatch.

When he was at the party, *the girl* cruelly teased the boy during the party games.

Their results showed that English native speakers exhibited longer reading times in gender-mismatch than gender-match conditions at the adverb region immediately following the first noun phrase. This suggested that English native speakers predictively assigned the cataphoric pronoun to the first noun phrase, and the gender-incongruency between the cataphoric pronoun and the first noun phrase caused reader’s processing difficulty. This processing difficulty was thus referred to as GMME effect (Gender-Mismatch effect). The presence of GMME effect indicated that readers were so eager to resolve the cataphoric pronoun that they predictively linked the cataphoric pronoun to the first syntactic position that could contain a potential antecedent even before the gender information of the potential antecedent became available. This searching process in cataphora resolution is much like the active search mechanism adopted in processing filler-gap dependencies (e.g., Crain and Fodor, 1985; Stowe, 1986; Frazier and Flores D’Arcais, 1989), for both parsers attempt to find an antecedent or gap at the earliest possible syntactic position in order to establish the dependencies as soon as possible, without considering the plausibility of the constituent at that position (Kazanina et al., 2007). On this basis, active search mechanism is also used to describe the process of actively searching for antecedents in cataphora resolution.

English native speakers initiate an active search for the antecedent after encountering a cataphoric pronoun. This finding gave rise to another research question, i.e., whether the search process in cataphora resolution proceeds unconstrained or is subject to some constraints. The Binding Theory proposed by Chomsky (1981) offers theoretical justification for the interpretation of pronouns. Of the three principles contained in the Binding Theory, what is relevant to the interpretation of cataphoric pronouns is Principle C whereby referring expressions cannot co-refer with noun phrases including pronouns that c-command them. In the context of cataphora, Principle C can be restated as cataphoric pronouns cannot establish coreference relations with noun phrases that are c-commanded by them. Naturally, Principle C is able to be used to test whether the active searching process in cataphora resolution is restricted by the syntactic constraint.

Kazanina et al. (2007) were the first to demonstrate that Principle C constrained English natives’ online processing of cataphoric pronouns. Results from Kazanina et al.’s (2007) study showed that significantly longer reading times in gender-mismatch than in gender-match conditions were only observed in no-constraint pairs. This suggested that English native speakers only tried to establish coreference relations between the cataphoric pronoun and the first potential antecedent in the no-constraint conditions, indicating that Principle C indeed constrained English native speakers’ online processing of cataphoric pronouns Later, Principle C’s constraint in cataphora resolution was also confirmed by other studies (Kazanina and Phillips, 2010; Clackson and Clahsen, 2011; Yoshida et al., 2014; Pablos et al., 2015).

The active searching for the antecedent in cataphora resolution was restricted not only by Principle C, but also by Principle B, the principle that regulates co-arguments of the same predicate cannot establish coreference relations. For instance, Kush and Dillon (2021) employed self-paced reading technique to test whether Principle B constrained English native speakers’ cataphora resolution.

(2) a. Constraint, gender-match/gender-mismatch

While (PRO) driving him/her to school on Friday, Christopher casually told Juan/Hannah that he would pick up everyone early for a surprise.

b. No-constraint, gender-match/gender-mismatch.

While (PRO) driving his/her daughter to school on Friday, Christopher casually told Juan/Hannah that he would pick up everyone early for a surprise.

They designed cataphoric pronouns to appear in a fronted participle clause, with its implicit subject obligatorily interpreted as the matrix subject according to Control Theory (Chomsky, 1981), consequently making the coreference between the cataphoric pronoun and the matrix subject syntactically-illicit, as illustrated in (2a). In (2b), by contrast, with the cataphoric pronoun being embedded inside the noun phrase *his/her daughter*, the implicit subject PRO and the cataphoric pronoun were no longer co-arguments of the same predicate, which allowed for coreference between PRO and the cataphoric pronoun, and further made the coreference between the cataphoric pronoun and matrix subject syntactically-licit. Kush and Dillon (2021)’s results showed that the GMME effect at the matrix subject region (Christopher) and its spillover region (casually) was only present in the no-constraint conditions, demonstrating that Principle B restricted English native speakers’ active searching for the cataphor antecedent.

In summary, cataphora resolution studies on native speakers have consistently shown that encountering a cataphoric pronoun triggers an active search for the antecedent and this searching process is restricted by syntactic constraints (Principle B and Principle C) such that only syntactically-licit noun phrases can be considered as antecedents of cataphoric pronouns.

Compared to cataphora resolution studies on native speakers, very little research has been conducted to investigate how L2 learners resolve cataphoric pronouns (Rodríguez, 2008; Bertenshaw, 2009; Drummer and Felser, 2018; Wu et al., 2019). Using eye-tracking technique, Drummer and Felser (2018) compared the online performance of German native speakers and Russian German learners in processing German cataphoric pronouns, revealing that Russian German learners behaved themselves just as German native speakers in using an active search mechanism that was constrained by Principle C. Similarly, Bertenshaw (2009) found that both English native speakers and Japanese English learners followed Principle C when resolving cataphoric pronouns online. Another comparative study using self-paced reading technique indicated that both English native speakers and Spanish English learners abided by Principle C in processing English cataphoric pronouns while the GMME effect restricted to no-constraint conditions did not reach statistical significance in Chinese English learners (Rodríguez, 2008). Wu et al. (2019), who adopted self-paced reading technique to compare Chinese EFL learners’ processing of forwards anaphora and cataphora, found that the GMME effect was not statistically significant in their processing of cataphoric pronouns.

Evidently, previous cataphora resolution studies on English native speakers have consistently shown that native speakers initiate an active search for the cataphor antecedent, in accordance with Principle B and C. Though a few studies on EFL learners revealed similar results, the learners have L1s basically similar to English in linguistic typology. It remains unknown how EFL learners with typologically distinct L1s like Chinese resolve English cataphora. Whether there exists cataphora in Chinese has been a controversial issue. Some scholars (Xu and He, 2007; Gao, 2010; Yu, 2011) hold that zero pronouns in the preposed (clause-initial) modifiers can form cataphoric relations with noun phrases which act as the main clause subjects or the modifiers of the main clause subjects. For example, Gao (2010) used the following sentences in (3) to exemplify his view that zero cataphoric pronouns exist in both Chinese and English and they are used in a similar way in the two languages. However, other scholars (Wang, 1994; Wang, 2000, 2006; Zhao and Shao, 2002) believes that third person pronouns in Chinese do not have the cataphoric function, as can be seen in (4). Combining the two views together, we can conclude that zero cataphoric pronouns are present in both Chinese and English while overt cataphoric pronouns exist only in English. Since there are no overt cataphoric pronouns in Chinese, it is more informative to investigate Chinese EFL learners’ online processing of English overt cataphoric pronouns.

(3) ∅_i_自得地点了点头之后，蒋老虎_i_关心地问…

When ∅_i_ introducing Huan-chih to the boys of course, Ping-ju_i_ was at greater pain to create a good impression.

(4) ^*^他_i_看见了小李_i_的妈妈。

Against the above-mentioned background, the current study was undertaken to explore how Chinese EFL learners resolve English cataphoric pronouns by two eye-tracking experiments. Unlike previous studies on cataphora resolution, we referred to Kush and Dillon (2021) to test whether Principle B would affect Chinese EFL learners’ online processing of cataphoric pronouns. This option arises from the theoretical assumption by which Principle B is more suitable than Principle C for testing the role of syntactic constraint in cataphora resolution, for the application of Principle C in cataphora resolution involves some semantic and pragmatic considerations (Reinhart, 1983; Levinson, 1991; Reinhart and Reuland, 1993; Huang, 1994; Huang, 2000; Büring, 2005) while the application of Principle B is purely syntactic. In addition, considering learners’ proficiency level plays an important role in L2 sentence processing, we included two groups of Chinese EFL learners (high-proficiency group and low-proficiency group, HG and LG henceforth), to explore how learners’ proficiency level might modulate their online processing of cataphoric pronouns.

Specifically, this study was conducted to investigate whether Chinese EFL learners with different proficiency levels would adopt the pattern (i.e., an active search mechanism) similar to English natives in English cataphora resolution and whether the cataphora processing would be modulated by Principle B. In association with previous studies, we made the following hypotheses corresponding to the two research questions.

Firstly, both HG and LG learners would adopt an active search mechanism like English natives when resolving cataphoric pronouns, as would be indicated by longer reading times at the critical word region or its spillover region in gender-mismatching than in gender-matching sentences (i.e., GMME effect). Besides, learners’ English proficiency discrepancy might modulate the processing time-course, i.e., HG learners could be earlier than LG learners in initiating the search mechanism, as would be indicated by the GMME effect being observed in both early and late eye-movement measures at the critical word region and its spillover region for HG learners while the GMME effect being found in only late eye-movement measures at the critical word region or its spillover region for LG learners.

Secondly, Chinese EFL learners would observe Principle B in processing English cataphora, as would be suggested by a GMME effect restricted to no-constraint sentences at the critical word region or its spillover region. Furthermore, the processing pattern might vary with the learners’ English proficiency as well. Specifically, HG learners might behave more like English natives, exhibiting early application of Principle B in cataphora resolution, as would be indexed by the GMME effect restricted to no-constraint sentences being found in both early and late eye-movement measures at the critical word region and its spillover region, while LG learners would show delayed application of Principle B, as would be evidenced by the GMME effect restricted to no-constraint sentences being found in only late eye-movement measures at the critical word region or its spillover region.

## 2. Experiment 1

Experiment 1 was conducted to explore whether Chinese EFL learners would adopt an active search mechanism to resolve cataphoric pronouns as English natives do, and how learners’ proficiency would modulate this cataphora resolution process.

### 2.1. Participants

Thirty-one English majors who had passed TEM-8 (Test for English Majors-Band 8) and thirty-two non-English majors who had scored between 430 and 480 on the CET-4 (College English Test Band 4) but had not passed the CET-6 (College English Test Band 6) were recruited as our participants. Of all the participants, the data of five participants (1 English majors and 4 non-English majors) were excluded from further analyses as their accuracy rate was below 60%. The data to enter into final analysis consisted of 30 English majors (age: *M =* 22 years, SD *=* 1.11 years, ranging from 20 years to 24 years; gender: 10 males, 20 females) and 28 non-English majors (age: *M =* 21 years, SD *=* 1.17 years, ranging from 19 years to 23 years; gender: 10 males, 18 females). Before the experiment, all the participants were required to complete an adapted version of language history questionnaire (Li et al., 2006) in which they needed to report their AoA (Age of Acquisition) of English and make a self-assessment on their English listening, speaking, reading and writing abilities (five points for full marks for each ability). Besides, they also needed to complete the Oxford Placement Test (QPT), a standardized test for English proficiency. Results of the questionnaire and QPT scores are given in Table 1.

**Table 1:** Results of the language history questionnaire and QPT scores in Experiment 1.

	HG (English majors)		LG (non-English majors)	
	*M*	SD	*M*	SD
**Self-rating**				
Listening	2.86	0.69	2.46	0.63
Speaking	3.23	0.63	2.40	0.75
Reading	3.77	0.55	3.12	0.80
Writing	3.19	0.74	2.77	0.85
AoA	9.75	1.97	8.88	2.01
QPT	53.10	1.77	36.18	1.68

English majors (*M* = 53.10, SD = 1.77) scored significantly higher than non-English majors (*M* = 36.18, SD = 1.68) in the QPT (*t* (56) = 37.312, *p <* 0.001). Two independent-sample *t*-test on participants’ total scores of four English abilities also showed that English majors (*M* = 13.05, SD = 1.86) scored significantly higher than non-English majors (*M* = 10.75, SD = 2.09; *t* (56) = −4.421, *p <* 0.001). At the same time, the self-rated score on English reading ability was positively correlated with the OPT score for both English majors (*r* (28) =0.817, *p <* 0.001) and non-English majors (*r* (26) = 0.899, *p <* 0.001). On account of this, we took English majors as HG learners and non-English majors as LG learners. Both HG and LG learners are late second language learners, with no significant difference in their AoA of English (HG: *M* = 9.75, SD = 1.97; LG: *M* = 8.88, SD = 2.01; *t* (56) = −1.651, *p =* 0.104). All participants were right-handed (Oldfield, 1971) native Chinese speakers with normal or corrected to normal vision and did not have any psychiatric or reading disorders. Each participant signed a formal inform consent before the experiment and were paid for their participation after the experiment. The experiment was approved by the Ethics Committee of Qufu Normal University.

### 2.2. Materials

Our stimuli consisted of 50 pairs of sentences as shown in (5). Of the 50 pairs of sentences, 24 pairs were adapted from the sentences in no-constraint conditions in Kush and Dillon (2021)’s Experiment 2, using familiar names and words to substitute for the corresponding unfamiliar counterparts, and the other 26 pairs were constructed by referring to the sentence structures in Kush and Dillon (2021). The 50 pairs of experimental sentences were selected from a total of 80 pairs of sentences. Thirty-four LG learners who did not participate in the formal experiment but were selected from non-English majors based on the same criteria as in formal experiment were recruited to rate the comprehensibility of all the sentences on a 5-point Likert scale (1 = totally incomprehensible, 5 = totally comprehensible). As a result, the sentences with over 3.75 rating score on average were chosen as the experimental stimuli (*M* = 4.33, SD = 0.31) so that all were equally understood by both HG and LG learners.

(5) a. gender-match
  After a stranger bought her an iced coffee, Linda generously offered Elsa the best seat at the meeting table.

b. gender-mismatch.

After a stranger bought him an iced coffee, Linda generously offered Victor the best seat at the meeting table.

As shown in (5), all the stimuli are made up of two parts, the subordinate clause (headed by *while*, *before* or *after*) as the first part and the main clause as the second part. The cataphoric pronoun occurs in the direct object position of the subordinate clause with an indefinite noun phrase (e.g., *a stranger*) or an indefinite pronoun (someone, anyone) as the subject. The main clause contains two names, one in subject position (followed by an adverb) and the other in object position. Adverbs following the matrix subject name were those with a weak pragmatic function, such as *casually*, *generously*, *kindly* and the like. All the adverbs were selected from the lexical syllabus for CET-4 and acquired an average rating score of more than 3.75 on a 5-ponit Likert scale (*M* = 4.01, SD = 0.58) in a familiarity pretest (1 = totally unfamiliar, 5 = totally familiar) by the same group of LG learners who participated in the sentence comprehensibility rating. The gender of the cataphoric pronoun was manipulated to either match or mismatch the gender of the matrix subject. The gender of the matrix object name was designed to match that of the cataphoric pronoun so that the pronoun could be resolved intra-sententially. All the sentences in gender-match conditions were counterbalanced so that the cataphoric pronouns *him* and *her* appeared evenly in the stimuli.

The experimental sentences were divided into two lists by a Latin-Square design, with 75 extra sentences as fillers which were similar to the target sentences in length and complexity, but did not contain cataphoric pronouns. To ensure that participants could attend to the reading task, 25 experimental sentences and 38 filler sentences were followed by a comprehension question for each. In case participants did not resolve cataphoric pronouns during reading, 7 out of the 25 comprehension questions following the experimental sentences required the interpretation of cataphoric pronouns (e.g., Who got an iced coffee?) and the remaining questions asked about argument roles in the main clause or subordinate clause (Who offered someone the best seat?/Who got the best seat?/Who bought someone an iced coffee?).

As illustrated in (5), four regions (underlined parts) were selected for experimental analyses: Region 1 (e.g., Linda), the main clause subject (the first potential antecedent of the cataphoric pronoun); Region 2 (e.g., generously), the adverb following the matrix subject, serving as the spillover region of Region 1; Region 3 (e.g., Elsa/Victor), the direct object in the main clause (the other potential antecedent for the cataphoric pronoun); Region 4 (e.g., the best), the spillover region of Region 3, containing two words immediately following Region 3.

### 2.3. Procedure

The whole experiment was carried out in a quiet laboratory room. Participants were seated ~75 cm away from the display screen and 60 cm away from the eye-tracker. The eye-movement data were recorded using the desk-mounted Eyelink 1000 plus eye-tracker (SR Research, Toronto, ON, Canada) with a sampling rate of 1,000 Hz and a refresh rate of 150 Hz. Right eye was recorded. All the experimental sentences were displayed in 19-point Consolas font across two lines on a 19-inch computer screen.

Each participant was randomly assigned to one of the two presentation lists in which sentences were presented in a pseudo-randomized order such that two sentences in the same condition would not appear more than two consecutive times. Before the formal experiment, a nine-point calibration procedure was performed, followed by a practice session of five sentences for each participant. Drift corrections were performed between trials. All participants were instructed to read the sentences in a natural speed. After finishing reading the sentences, they should press the space key to answer questions or move into next trial. Once a question sentence appeared, participants should press either “F” or “J” to make a response, with the two keys counterbalanced across participants. The whole experiment was comprised of two sessions with a short interval in between. The whole experiment lasted about 40–50 min for HG learners and 60–70 min for LG learners.

### 2.4. Data analysis

Following Drummer and Felser (2018), we reported four eye-movement measures in each of the four regions: First Fixation Duration (FFD), First Pass Reading Time (FPRT), Regression Path Duration (RPD) and Total Reading Time (TRT).

Before exporting the eye-movement data, fixations that were shorter than 80 ms but within one character space of the previous or next fixations were merged with their neighboring fixations. The remaining fixations that were shorter than 80 ms or longer than 800 ms were excluded because they could not reflect proper language processing (Rayner and Pollatsek, 1989; Rayner, 1998). For each eye-movement measure, the data above or below three standard deviations were removed from further analyses. For HG learners, this accounted for 5.76% of the data in the by-participant analysis and 6.12% of the data in the by-item analysis. For LG learners, this accounted for 4.91% of the data in the by-participant analysis and 4.57% of data in the by-item analysis.

Two-way mixed repeated measures ANOVAs with participants (*F1*) and items (*F2*) as random variables were carried out for each eye-movement measure with between-subject factor Group (HG vs. LG) and within-subject factor Gender Match (yes vs. no) using SPSS Statistics 26.0. Table 2 displays two participant groups’ mean reading times on the four regions per condition and Table 3 provides a summary of ANOVAs for each region.

**Table 2:** Mean reading times in milliseconds by region in Experiment 1 (SDs in parentheses).

		FFD		FPRT		RPD		TRT	
Region	Condition	High	Low	High	Low	High	Low	High	Low
1	Match	235 (22)	234 (31)	394 (50)	436 (87)	409 (58)	471 (94)	1,011 (239)	1,228 (315)
	Mismatch	229 (29)	237 (30)	373 (55)	450 (86)	394 (64)	476 (98)	947 (243)	1,264 (295)
2	Match	252 (38)	271 (28)	362 (57)	466 (81)	557 (108)	698 (123)	990 (239)	1,353 (370)
	Mismatch	249 (40)	265 (25)	343 (61)	472 (79)	579 (121)	674 (139)	951 (209)	1,305 (323)
3	Match	262 (33)	273 (29)	312 (42)	361 (58)	2,580 (865)	332 (1182)	990 (254)	1,185 (352)
	Mismatch	251 (29)	275 (34)	301 (40)	345 (41)	2,361 (920)	316 (1162)	915 (257)	1,099 (342)
4	Match	234 (26)	252 (21)	362 (45)	452 (62)	2,875 (989)	3,661 (1,342)	765 (168)	1,003 (269)
	Mismatch	235 (27)	249 (20)	370 (55)	447 (63)	2,640 (1043)	3,661 (1,445)	751 (171)	957 (208)

**Table 3:** Summary of ANOVAs for each region in Experiment 1.

	Region 1		Region 2		Region 3		Region 4	
	*F1*(1, 56)	*F2*(1, 98)	*F1*(1, 56)	*F2*(1, 98)	*F1*(1, 56)	*F2*(1, 98)	*F1*(1, 56)	*F2*(1, 98)
**FFD**								
Group	0.244	0.752	4.506^*^	21.369^***^	5.442^*^	20.414^***^	7.360^**^	31.336^***^
Match	0.175	0.655	1.930	1.630	1.602	2.062	0.354	0.153
Gr*Mat	2.296	2.155	0.112	0.339	3.533^(*)^	2.366	0.789	0.641
**FPR**								
Group	11.314^**^	37.404^***^	46.614^***^	75.533^***^	18.269^***^	55.202^***^	40.676^***^	26.748^***^
Match	0.475	0.429	1.146	0.725	8.003^**^	4.876^*^	0.034	0.220
Gr*Mat	12.626^**^	9.568^**^	3.647^(*)^	1.495	0.275	0.336	0.835	0.247
**RPD**								
Group	12.865^**^	41.914^***^	16.307^***^	29.222^***^	8.580^**^	133.969^***^	8.645^**^	96.993^***^
Match	0.801	1.073	0.002	0.058	12.203^**^	8.708^**^	2.985	2.566
Gr*Mat	3.468^(*)^	2.242	2.789	2.702	0.280	0.501	2.952	3.066
**TRT**								
Group	14.549^***^	61.266^***^	24.397^***^	150.809^***^	6.051^*^	51.642^***^	19.181^***^	32.503^***^
Match	0.732	0.552	4.494^*^	3.542^(*)^	19.784^***^	13.362^***^	2.569	3.058
Gr*Mat	9.891^**^	8.082^**^	0.046	0.018	0.087	0.000	0.777	0.903

(*)*p* < 0.1; *p < 0.05; **p < 0.01; ***p < 0.001.

### 2.5. Results

#### 2.5.1. Behavioral data

The mean accuracy was 81.38% (SD = 0.10) for HG learners and 71.38% (SD = 0.09) for LG learners, which were both significantly above the chance level (HG: *t* (29) = 17.960, *p <* 0.001; LG: *t* (27) = 12.475, *p <* 0.001), showing that all the participants were attentive to the reading task.

#### 2.5.2. Eye-tracking data

The following is the statistical results of the eye-tracking data in association with each region. By convention, only the significant results are reported as below. All significant and non-significant results may be seen at Table 3.

##### 2.5.2.1. Region 1

For first fixation durations, no significant main effects or interactions were found (*ps >* 0.1).

For first pass reading times, a significant interaction between Gender Match and Group was found (*p1 =* 0.001; *p2 = 0*.003). Simple effects analysis revealed that only HG learners spent significantly longer reading times in gender-match than in gender-mismatch conditions (*F1*(1, 56) = 10.039, *p =* 0.002; *F2*(1, 98) = 7.025, *p = 0*.009).

For regression path durations, a marginally significant interaction between Gender Match and Group in the participant analysis was observed (*p1 =* 0.068; *p2 = 0*.137). Simple effects analysis showed that only HG learners exhibited significantly longer regression path durations in gender-match than in gender-mismatch conditions (*F1*(1, 56) = 4.239, *p =* 0.044; *F2*(1, 98) = 3.209, *p = 0*.076).

For total reading times, a significant interaction between Gender Match and Group was found (*p1* = 0.003; *p2 =* 0.005). Simple effects analysis showed that only HG learners demonstrated significantly longer total reading times in gender-match than in gender-mismatch conditions (*F1*(1, 56) = 8.926, *p =* 0.004; *F2*(1, 98) = 6.428, *p = 0*.013).

##### 2.5.2.2. Region 2

For first fixation durations, no significant main effects or interactions were found (*ps >* 0.1).

For first pass reading times, a marginally significant interaction between Gender Match and Group in the participant analysis was observed (*p1 =* 0.061; *p2 =* 0.224). Simple effects analysis showed that only HG learners spent longer reading times in gender-match than in gender-mismatch conditions (*F1*(1, 56) = 4.954, *p =* 0.030; *F2*(1, 98) = 2.152, *p =* 0.146).

For regression path durations, no significant main effects or interactions were found (*ps >* 0.1).

For total reading times, a significant main effect of Gender Match in the participant analysis was observed (*p1 =* 0.038; *p2 =* 0.063). Both HG and LG learners spent significantly longer reading times in gender-match than in gender-mismatch conditions.

##### 2.5.2.3. Region 3

For first fixation durations, a marginally significant interaction between Gender Match and Group in the participant analysis was found (*p1 = 0.*065; *p2 =* 0.127). Simple effects analysis showed that only in gender-mismatch conditions, did LG learners exhibit longer first fixation durations than HG learners (*F1*(1, 56) = 8.237, *p = 0.*006; *F2*(1, 98) = 21.842, *p <* 0.001). Meanwhile, HG learners spent longer first fixation durations in gender-match than in gender-mismatch conditions (*F1*(1, 56) = 5.517, *p = 0.*022; *F2*(1, 98) = 4.423, *p =* 0.038).

For first pass reading times, a significant main effect of Gender Match was found (*p1 =* 0.006; *p2 =* 0.030). First pass reading times in gender-match conditions were significantly longer than those in gender-mismatch conditions for both HG learners and LG learners.

For regression path durations, a significant main effect of Gender Match was found (*p1 =* 0.001; *p2 =* 0.004). Both HG and LG learners showed significantly longer reading times in gender-match than in gender-mismatch conditions.

For total reading times, a significant main effect of Gender Match was observed (*p1 <* 0.001; *p2 <* 0.001). Both HG and LG learners spent significantly longer reading times in gender-match than in gender-mismatch conditions.

##### 2.5.2.4. Region 4

For all eye-movement measures (first fixation durations, first pass reading times, regression path durations, total reading times), no significant main effects or interactions were observed at this region (ps > 0.05).

### 2.6. Discussion

Experiment 1 intended to explore whether Chinese EFL learners initiated an active searching process in cataphora resolution and whether learners’ proficiency level influenced this searching process. The eye-movement data revealed a GME effect (Gender Match Effect) for all participants, but the effect was observed earlier in HG learners than in LG learners.

Contrary to a GMME effect that has been consistently observed in previous studies on cataphora resolution (van Gompel and Liversedge, 2003; Kazanina et al., 2007; Kazanina and Phillips, 2010; Clackson and Clahsen, 2011; Yoshida et al., 2014; Pablos et al., 2015; Patterson and Felser, 2019), our study unexpectedly revealed a GME effect at region 1 (subject name) and region 2 (adverb), suggesting that a gender-matching potential antecedent elicited longer reading times than its gender-mismatching counterpart at the matrix subject position. GMME effect has been taken as an indication of readers’ employment of active search mechanism in cataphora resolution, so did the absence of GMME effect mean Chinese EFL learners did not resolve the cataphoric pronoun in an active way? It seems hasty to jump to such a strong conclusion. Since readers’ active search for the cataphor’s antecedent implies a link has already been established between the cataphoric pronoun and the first syntactic position that could contain a noun phrase even before the information (e.g., gender and number) about the noun phrase is accessed, the nature of the active search mechanism lies in whether readers predictively link a cataphoric pronoun to the first syntactic position that could contain a potential antecedent. Based on this rationale, we believe that Chinese EFL learners were also engaged in an active search for the antecedent in the cataphora resolution process, otherwise the gender features of the first potential antecedent would not affect their reading times (though in a different direction from English natives). However, this active searching process adopted by Chinese EFL learners was distinct from the one used by English natives in that only a gender-matching antecedent could prompt Chinese EFL learners to engage in a deep processing of this antecedent, contrasting with English natives’ deep processing of both gender-matching and gender-mismatching antecedents, consequently resulting in GME effect for Chinese EFL learners while GMME effect for English natives. As for the reason why Chinese EFL learners behaved differently from English native speakers when facing a gender-mismatching name at the matrix subject position, we will elaborate it in the General Discussion part.

Consistent with our predictions, LG learners were found to behave differently from HG learners in the processing of cataphoric pronouns. HG learners exhibited a GME effect in first pass reading times, regression path durations and total reading times at region 1, while LG learners only showed a GME effect in total reading times at region 2, i.e., gender features of subject names did not affect LG learners’ processing time until region 2 (the spillover region of the subject name). This suggests that LG learners, relative to HG learners, delayed their active searching process in cataphora resolution.

Significantly longer reading times in gender-match than in gender-mismatch conditions were also observed in first pass reading times, regression path durations and total reading times at region 3 (object name region). However, the gender-match effect at region 3 was different from that at region 1 and region 2. The name at region 3 provides another antecedent candidate in gender-match conditions while it is the only suitable antecedent in gender-mismatch conditions. Therefore, the longer reading times observed in gender-match than in gender-mismatch conditions might due to the competition between two gender-matching names for acting as the antecedent of the cataphoric pronoun. In addition, it appeared that HG learners were earlier influenced by the competition between subject name and object name in gender-match conditions as indicated by longer first fixation durations in gender-match conditions observed only for HG learners.

In summary, the results of Experiment 1 suggest that Chinese EFL learners also adopt an active search mechanism to resolve cataphoric pronouns, yet along a processing path distinct from English natives’. Specifically, Chinese EFL learners predictively link a cataphoric pronoun to the first potential antecedent in the sentence but only a gender-matching antecedent can prompt them to engage in deep processing of the antecedent. Moreover, the learners’ proficiency level modulates the time-course of their active searching process, with HG learners initiating an active search for the antecedent earlier than LG learners.

Both HG and LG Chinese EFL learners initiate an active search for the antecedent after encountering a cataphoric pronoun. Whether this searching process is also constrained by Binding Principles in the same way as English natives has not been clear, the issue Experiment 2 was to explore.

## 3. Experiment 2

Experiment 1 has shown that Chinese EFL learners initiated an active search for the cataphor’s antecedent. Experiment 2 was thus conducted to further investigate whether Chinese EFL learners’ active searching process is governed by the Binding Principle B. If so, we also want to explore whether learners’ proficiency influences the time-course of the application of Binding Principle B.

### 3.1. Participants

The recruitment criteria for participants were the same as in Experiment 1. A total of 27 English majors and 30 non-English majors participated in this experiment. The data of three participants (all non-English majors) were discarded as their accuracy rate was below 60%. The data for final analysis comprised of 27 English majors (age: *M =* 22.17 years, SD *=* 1.26 years, ranging from 20 years to 26 years; gender: 9 males, 18 females) and 27 non-English majors (age: *M =* 20.88 years, SD *=* 1.19 years, ranging from 19 years to 23 years; gender: 10 males, 17 females). All participants completed an adapted version of language history questionnaire (Li et al., 2006) and the QPT before the experiment. The results of the language history questionnaire and QPT scores are provided in Table 4.

**Table 4:** Results of the language history questionnaire and QPT scores in Experiment 2.

	HG (English majors)		LG (non-English majors)	
	*M*	SD	*M*	SD
**Self-rating**				
Listening	3.11	0.76	2.44	0.80
Speaking	3.10	0.87	2.48	0.76
Reading	3.90	0.56	3.35	0.85
Writing	3.31	0.72	2.96	0.95
AoA	8.80	1.90	9.38	1.74
QPT	52.37	1.52	35.81	1.75

As in Experiment 1, English majors (*M* = 52.37, SD = 1.52) were more proficient than non-English majors (*M* = 35.81, SD = 1.75; *t* (52) = 37.023, *p <* 0.001), which was also confirmed by the results from participants’ total scores of four English abilities, showing that English majors (*M* = 13.41, SD = 2.02) scored significantly higher than non-English majors (*M* = 11.22, SD = 2.68; *t*(52) = −3.433, *p* = 0.001). Similarly, self-rated score on English reading ability was positively correlated with the OPT score for both English majors (*r* (25) = 0.749, *p <* 0.001) and non-English majors (*r* (25) = 0.863, *p <* 0.001). Therefore, we grouped English majors as HG learners and non-English majors as LG learners. Both two participant groups were late second language learners, with no significant difference in their AoA of English (HG: *M* = 8.80 years, SD = 1.90 years; LG: *M* = 9.38 years, SD = 1.74 years; *t* (52) =1.146, *p =* 0.257). All participants signed a written inform consent before the experiment and were paid for their participation after the experiment.

### 3.2. Materials

The materials consisted of 60 sets of sentences as shown in (6). Each sentence was made up of one main clause and one fronted participle clause headed by *while*, *before* or *after*. All the materials were constructed following a two-factors (Constraint and Gender-Match) within-subjects design. The factor Constraint manipulated whether the coreference between the cataphoric pronoun and the matrix subject was constrained by Binding Principle B. In constraint conditions, the cataphor was the direct object of the infinitival verb while in no-constraint conditions, the cataphor was a possessive pronoun embedded in the direct object noun phrase. Across constraint and no-constraint conditions, the gender of the cataphoric pronoun was manipulated to either match or mismatch the gender of the matrix subject. As in Experiment 1, all the adverbs following the matrix subjects were selected from the lexical syllabus for CET-4 and met the requirement of an average rating score of more than 3.75 on a 5-ponit Likert scale (*M* = 4.12, SD = 0.46) in a familiarity pretest (1 = totally unfamiliar, 5 = totally familiar) by an additional group of 60 LG learners who were selected from non-English majors based on the same criteria as in formal experiment. There was a gender-matching name at the matrix object position across four conditions to ensure that each cataphoric pronoun was to have an intra-sentential referent.

Of the 60 sets of target sentences, 24 sets were adapted from Kush and Dillon (2021)’s Experiment 1 and the other 26 sets were constructed following the same sentence patterns. The 60 sets of target sentences were selected from a total of 85 sets of sentences. The same group of LG learners who participated in the familiarity test on adverbs were also asked to rate the comprehensibility of those sentences on a 5-point Likert scale (1 = totally incomprehensible, 5 = totally comprehensible). Sentences with 3.75 rating score on average were selected as the experimental items (*M* = 4.34, SD = 0.28).

(6) a. constraint, gender-match

After buying her an iced coffee, Linda generously offered Elsa the best seat at the meeting table.

b. constraint, gender-mismatch.

After buying him an iced coffee, Linda generously offered Victor the best seat at the meeting table.

c. no-constraint, gender-match.

After buying her colleague an iced coffee, Linda generously offered Elsa the best seat at the meeting table.

d. no-constraint, gender-mismatch.

After buying his colleague an iced coffee, Linda generously offered Victor the best seat at the meeting table.

To ensure that each participant would receive a sufficient number of target sentences from each condition, the 60 sets of experimental sentences were divided into two lists in such a way that each participant could see two sentences from one set, one in constraint condition, and the other in no-constraint condition. Each list consisted of 120 experimental sentences (30 experimental sentences for each condition) and mixed with equal 120 fillers. As in Experiment 1, the fillers were of similar length and complexity to the experimental sentences and did not contain cataphoric pronouns. Similarly, half of the experimental sentences and filler sentences were followed by a comprehension question. In case participants did not resolve cataphoric pronouns during reading, 12 out of the 60 comprehension questions following the experimental sentences required the interpretation of cataphoric pronouns (e.g., Who got an iced coffee?/Whose colleague got an iced coffee?) and the remaining questions mainly targeted the interpretation of argument roles in the main clause (e.g., Who offered someone the best seat?/Who got the best seat?) or the interpretation of the implicit subject in the adjunct clause (e.g., Who bought someone an iced coffee?).

Also similar to Experiment 1, four regions were selected for further analyses. Region 1 was the matrix subject name; Region 2 was the adverb following the matrix subject; Region 3 was the matrix object name; Region 4 consisted of two words that came after Region 3.

### 3.3. Procedure

The procedure was identical to Experiment 1. The formal part of the experiment was divided into three blocks with a short break in between. After each break, a nine-point calibration procedure was conducted again. All the stimuli in each presentation list were presented in a pseudo-randomized order such that two sentences in the same condition would not consecutively appear more than twice. The entire experiment lasted about 70–80 min for HG learners and 80–90 min for LG learners.

### 3.4. Data analysis

The same four eye-movement measures were reported as in Experiment 1: first fixation duration, first pass reading time, regression path duration and total reading time.

Before exporting the eye-movement data, fixations shorter than 80 ms but within one character space of the previous or next fixations were merged with their neighboring fixations. The fixations that were shorter than 80 ms or longer than 800 ms were excluded as well. For each eye-movement measure, the data that were above or below three standard deviations were discarded from further analyses. For HG learners, this accounted for 7.90% of data in the by-participant analysis and 5.66% of data in the by-item analysis. For LG learners, this accounted for 6.14% of the data in the by-participant analysis and 6.28% of data in the by-item analysis.

Three-way mixed repeated measures ANOVAs with participants (*F1*) and items (*F2*) as random variables were carried out for each eye-movement measure with between-subject factor Group (HG vs. LG) and within-subject factors Constraint (yes vs. no) and Gender Match (yes vs. no) using SPSS Statistics 26.0. Table 5 displays two participant groups’ mean reading times on the four regions per condition and Table 6 provides a summary of ANOVAs for each region.

**Table 5:** Mean reading times in milliseconds by region in Experiment 2 (SDs in parentheses).

Region	Condition		FFD		FPRT		RPD		TRT	
			High	Low	High	Low	High	Low	High	Low
1	Constraint	Match	247 (26)	248 (25)	363 (67)	426 (57)	377 (72)	477 (71)	858 (172)	1,359 (283)
		Mismatch	240 (24)	250 (34)	355 (62)	438 (67)	369 (65)	469 (64)	816 (164)	1,339 (237)
	No-constraint	Match	234 (24)	237 (24)	348 (61)	433 (52)	360 (63)	475 (57)	857 (187)	1,421 (281)
		Mismatch	238 (20)	246 (30)	351 (59)	433 (46)	368 (64)	470 (52)	849 (168)	1,413 (263)
2	Constraint	Match	253 (28)	285 (37)	308 (47)	413 (65)	412 (72)	646 (92)	669 (142)	1,179 (190)
		Mismatch	260 (28)	291 (31)	320 (35)	437 (77)	401 (62)	611 (106)	634 (118)	1,176 (207)
	No-constraint	Match	251 (28)	274 (25)	308 (41)	403 (66)	435 (77)	635 (92)	727 (165)	1,191 (201)
		Mismatch	254 (28)	283 (27)	316 (46)	408 (61)	444 (74)	658 (111)	694 (108)	1,234 (212)
3	Constraint	Match	254 (19)	284 (18)	299 (32)	361 (46)	1,928 (606)	3,585 (871)	867 (257)	1,377 (310)
		Mismatch	259 (22)	283 (24)	298 (33)	357 (41)	1,792 (582)	3,455 (763)	783 (223)	1,336 (302)
	No-constraint	Match	247 (17)	286 (20)	291 (29)	365 (40)	2,063 (679)	3,676 (813)	847 (251)	1,439 (312)
		Mismatch	255 (26)	279 (23)	292 (30)	362 (47)	1,941 (564)	3,780 (772)	860 (247)	1,505 (319)
4	Constraint	Match	232 (18)	258 (17)	318 (36)	439 (70)	2,101 (645)	3,936 (991)	546 (98)	929 (198)
		Mismatch	238 (21)	248 (17)	344 (45)	442 (67)	1,945 (614)	3,887 (894)	533 (88)	964 (195)
	No-constraint	Match	234 (19)	256 (23)	332 (42)	442 (79)	2,250 (680)	4,182 (970)	587 (103)	1,007 (198)
		Mismatch	237 (20)	252 (21)	331 (46)	443 (62)	2,170 (639)	4,260 (937)	563 (94)	1,016 (193)

**Table 6:** Summary of ANOVAs for each region in Experiment 2.

	Region 1		Region 2		Region 3		Region 4	
	*F1*(1, 52)	*F2*(1, 118)	*F1*(1, 52)	*F2*(1, 118)	*F1*(1, 52)	*F2*(1, 118)	*F1*(1, 52)	*F2*(1, 118)
**FFD**								
Group	0.810	13.654^***^	17.574^***^	123.271^***^	42.892^***^	129.505^***^	16.791^***^	78.652^***^
Constraint	15.444^***^	11.306^**^	8.377^**^	6.965^**^	4.826^*^	2.618	0.188	0.025
Match	1.107	1.867	6.576^*^	7.274^**^	0.256	0.405	0.454	0.010
Gr × Cons	0.032	0.030	1.110	0.719	1.919	0.755	0.000	0.024
Gr × Mat	2.678	4.146^*^	0.082	0.095	4.578^*^	5.382^*^	8.523^**^	6.889^*^
Cons×Mat	4.924^*^	4.353^*^	0.004	0.091	0.142	0.060	0.054	0.516
Gr × Cons×Mat	0.391	0.143	0.684	0.669	1.043	1.059	2.611	3.569
**FPR**								
Group	27.342^***^	183.765^***^	57.322^***^	193.050^***^	56.992^***^	250.599^***^	66.110^***^	143.287^***^
Constraint	1.158	0.817	7.223^*^	2.630	0.251	0.127	0.155	0.031
Match	0.202	0.337	15.617^***^	9.231^**^	0.280	0.004	2.346	1.011
Gr × Cons	2.108	0.826	5.050^*^	1.612	4.825^*^	1.289	0.027	0.002
Gr × Mat	1.316	2.029	0.679	0.256	0.175	0.000	1.469	2.779
Cons×Mat	0.009	0.002	2.040	1.704	0.069	0.016	3.195	2.825
Gr × Cons×Mat	2.499	1.443	0.773	0.412	0.022	0.023	2.184	2.535
**RPD**								
Group	41.694^***^	195.344^***^	118.234^***^	223.450^***^	86.139^***^	508.318^***^	89.355^***^	476.871^***^
Constraint	1.424	0.641	17.116^***^	3.538^(*)^	21.034^***^	10.833^**^	35.765^***^	15.824^***^
Match	1.064	0.385	0.158	0.042	3.742	2.803	1.703	3.304
Gr × Cons	1.004	0.509	1.576	0.363	0.746	0.518	2.154	1.009
Gr × Mat	0.902	0.151	0.091	0.143	2.493	2.334	2.801	1.001
Cons×Mat	1.310	1.461	6.619^*^	3.782^(*)^	3.802	1.562	1.856	1.284
Gr × Cons×Mat	0.769	0.281	1.448	0.268	3.035	1.898	0.119	0.291
**TRT**								
Group	89.424^***^	486.376^***^	153.063^***^	295.218^***^	65.583^***^	418.565^***^	131.622^***^	227.672^***^
Constraint	14.846^***^	4.093^*^	16.641^***^	5.042^*^	24.496^***^	10.526^**^	20.181^***^	4.518^*^
Match	2.778	2.623	0.377	0.132	0.581	0.386	0.027	0.343
Gr × Cons	5.673^**^	2.089	1.086	0.187	8.825^**^	3.910^(*)^	1.763	0.331
Gr × Mat	0.246	0.102	5.334^*^	4.034^*^	2.501	2.115	3.790	0.762
Cons×Mat	1.153	0.541	1.148	0.612	11.041^**^	9.441^**^	1.121	0.099
Gr × Cons×Mat	0.281	0.111	0.955	0.357	0.025	0.042	0.174	0.055

(*)*p* < 0.1; ^*^p < 0.05; ^**^p < 0.01; ^***^p < 0.001.

### 3.5. Results

#### 3.5.1. Behavioral data

The mean accuracy was 87.28% (SD = 0.06) for HG learners and 80.83% (SD = 0.06) for LG learners, which were both significantly above the chance level (*t*(29) = 32.543, *p <* 0.001; *t*(23) = 24.147, *p <* 0.001), showing that all the learners completed the reading task attentively.

#### 3.5.2. Eye-tracking data

The significant statistical results of eye-tracking measurement are reported as below for each region. All significant and non-significant results may be seen at Table 6.

##### 3.5.2.1. Region 1

For first fixation durations, a significant main effect of Constraint was found (*p1 < 0.*001; *p2* = 0.001). Matrix subject names in constraint conditions required significantly longer first fixations than those in no-constraint conditions. A significant interaction was observed between Gender Match and Group in the item analysis (*p1 =* 0.108; *p2* = 0.044). Simple effects analysis revealed that LG learners spent significantly longer fixations than HG learners only in the gender-mismatch conditions (*F1*(1, 52) = 1.767, *p =* 0.190; *F2*(1, 118) = 14.765, *p < 0.*001). Besides, first fixation durations were significantly longer in gender-mismatch than in gender-match conditions but only occurred for LG learners (*F1*(1, 52) = 3.253, *p =* 0.077; *F2*(1, 118) = 5.789, *p = 0.*018). In addition, a significant interaction between Constraint and Gender Match was observed in first fixation durations (*p1 =* 0.031; *p2 =* 0.039). Simple effects analysis revealed that significantly longer first fixation durations in gender-mismatch than in gender-match conditions appeared only for no-constraint conditions (*F1*(1, 52) = 5.747, *p =* 0.020; *F2*(1, 118) = 5.545, *p =* 0.020) and significantly longer first fixation durations in constraint conditions than in no-constraint conditions appeared only in gender-match conditions (*F1*(1, 52) = 19.276, *p < 0.*001; *F2*(1, 118) = 17.286, *p < 0.*001).

For first pass reading times and regression path durations, no significant main effects or interactions were found (*ps* > 0.05).

For total reading times, a significant main effect of Constraint was found (*p1 < 0.*001; *p2* = 0.045). Reading times were significantly longer in no-constraint conditions than in constraint conditions. There was also a significant interaction between Constraint and Group in the participant analysis (*p1 =* 0.021; *p2 =* 0.151). Simple effects analysis showed that only LG learners spent significantly longer reading times in no-constraint than in constraint conditions (*F1*(1, 52) = 17.493, *p < 0.*001; *F2*(1, 118) = 6.015, *p =* 0.016).

##### 3.5.2.2. Region 2

For first fixation durations, a significant main effect of Constraint was found (*p1 =* 0.006; *p2 =* 0.009). Significantly longer fixations were observed in constraint than in no-constraint conditions. A significant main effect of Gender Match was also found (*p1 =* 0.013; *p2 =* 0.008). First fixation durations in gender-mismatch conditions were significantly longer than those in gender-match conditions.

For first pass reading times, a significant main effect of Constraint was found in the participant analysis (*p1 =* 0.010; *p2 =* 0.108). Reading times were significantly longer in constraint conditions than in no-constraint conditions. There was a significant main effect of Gender Match (*p1 <* 0.001; *p2 =* 0.003). Reading times in gender-mismatch conditions were significantly longer than those in gender-match conditions. Besides, a significant interaction between Constraint and Group was observed in the participant analysis (*p1 =* 0.029; *p2 =* 0.207). Simple effects analysis suggested that only LG learners exhibited significantly longer reading times in constraint than in no-constraint conditions (*F1*(1, 52) = 10.958, *p =* 0.002; *F2*(1, 118) = 4.180, *p =* 0.043).

For regression path durations, a significant main effect of Constraint was found in the participant analysis (*p1 <* 0.001; *p2 =* 0.062). Significantly longer regression times were elicited in no-constraint conditions than in constraint conditions. There occurred a significant interaction between Constraint and Gender Match (*p1 =* 0.013; *p2 =* 0.054). Simple effects analysis revealed that significantly longer regression path durations were observed in no-constraint than in constraint conditions but only for gender-mismatch conditions (*F1*(1, 52) = 20.457, *p < 0.*001; *F2*(1, 118) = 6.538, *p =* 0.012).

For total reading times, a significant main effect of Constraint was found (*p1 <* 0.001; *p2 =* 0.027). Reading times were significantly longer in no-constraint conditions than in constraint conditions. A significant interaction between Gender Match and Group was also found (*p1 =* 0.025; *p2 =* 0.047). Simple effects analysis showed that only HG learners demonstrated significantly longer reading times in gender-match than in gender-mismatch conditions (*F1*(1, 52) = 4.808, *p =* 0.033; *F2*(1, 118) = 2.812, *p =* 0.096).

##### 3.5.2.3. Region 3

For first fixation durations, a significant main effect of Constraint in the participant analysis was found (*p1 =* 0.033; *p2 =* 0.108). First fixations were significantly longer in constraint than in no-constraint conditions. There was also a significant interaction between Gender Match and Group (*p1 =* 0.037; *p2 =* 0.022). Simple effects analysis showed that only HG learners spent significantly longer fixations in gender-mismatch than in gender-match conditions (*F1*(1, 52) = 3.937, *p =* 0.053; *F2*(1, 118) = 4.369, *p =* 0.039).

For first pass reading times, a significant interaction between Constraint and Group was found in the participant analysis (*p1 =* 0.033; *p2 =* 0.259). Simple effects analysis showed that only HG learners spent significantly longer reading times in constraint than in no-constraint conditions (*F1*(1, 52) = 4.092, *p =* 0.048; *F2*(1, 118) = 1.112, *p =* 0.294).

For regression path durations, a significant main effect of Constraint was found (*p1 <* 0.001; *p2 =* 0.001). Regression path durations were significantly longer in no-constraint than in constraint conditions.

For total reading times, a significant main effect of Constraint was found (*p1 < 0.*001; *p2* = 0.002). Reading times were significantly longer in no-constraint conditions than in constraint conditions. There appeared a significant interaction between Constraint and Group (*p1 =* 0.004; *p2 =* 0.050). Simple effects analysis revealed that only LG learners spent significantly longer reading times in no-constraint than in constraint conditions (*F1*(1, 52) = 28.228, *p <* 0.001; *F2*(1, 118) = 13.633, *p < 0.*001). Furthermore, a significant interaction between Constraint and Gender Match was found (*p1 =* 0.002; *p2 =* 0.003). Simple effects analysis showed that significantly longer reading times were observed in gender-match than in gender-mismatch conditions but only for constraint conditions (*F1*(1, 52) = 8.941, *p =* 0.004; *F2*(1, 118) = 7.004, *p =* 0.009) while significantly longer reading times were observed in no-constraint than in constraint conditions but only for gender-mismatch conditions (*F1*(1, 52) = 28.268, *p < 0.*001; *F2*(1, 118) = 22.409, *p < 0.*001).

##### 3.5.2.4. Region 4

For first fixation durations, a significant interaction between Gender Match and Group was found (*p1 =* 0.005; *p2 =* 0.010). Simple effects analysis revealed that only LG learners spent significantly longer first fixations in gender-match than in gender-mismatch conditions (*p1 =* 0.020; *p2 =* 0.057).

For first pass reading times, no significant main effects or interactions were found (*ps* > 0.05).

For regression path durations, a significant main effect of Constraint was found (*p1 <* 0.001; *p2 < 0*.001). Regression path durations were significantly longer in no-constraint than in constraint conditions.

For total reading times, a significant main effect of Constraint was observed (*p1 <* 0.001; *p2 =* 0.036). Total reading times were significantly longer in no-constraint than in constraint conditions.

### 3.6. Discussion

Experiment 2 was conducted to further examine whether Chinese EFL learners’ cataphora resolution process was restricted by the syntactic constraint Binding Principle B and whether the processing pattern varied with the learners’ proficiency. Interpretations of the major results are presented in the following.^1^

Main effects of Gender Match were found for early eye-movement measures (first fixation durations and first pass reading times) at region 2, the spillover region of region 1. A gender-mismatching subject name elicited significantly longer reading times than a gender-matching one in both constraint pair and no-constraint pair. This GMME effect unaffected by the factor Constraint suggested that Chinese EFL learners tried to establish coreference relations between the cataphoric pronoun and the matrix subject in both constraint and no-constraint conditions, even if the coreference in constraint conditions violated Binding Principle B. The expected interaction between Constraint and Gender Match, however, just occurred in the late eye-movement measure (regression path durations) at region 2. A gender-mismatching subject induced more regressions in no-constraint conditions than in constraint conditions. In other words, the gender-incongruency between the cataphoric pronoun and the matrix subject only induced greater processing difficulty when the coreference between the cataphoric pronoun and the matrix subject was free from the constraint of Binding Principle B, suggesting that syntactically-illicit subject in constraint conditions was no longer considered as the potential antecedent of the cataphoric pronoun. Therefore, the early and late eye-tracking data at region 2 together demonstrated that Binding Principle B did not constrain Chinese EFL learners’ early processing of cataphoric pronouns, but only played its role in the late stage of cataphora resolution. Meanwhile, Chinese EFL learners’ delayed application of Binding Principle B also came up in the different effects of Constraint on first fixation durations and total reading times at region 2. While the main effect of Constraint on first fixation durations indicated that learners had more difficulty in processing constraint sentences, the main effect of Constraint on total reading times at the same region showed that the learners had more difficulty with no-constraint sentences. This indicates that in early processing, Chinese EFL learners were considering a potential antecedent which should be ruled out by Binding Principle B, but in later processing, they only considered a syntactically-licit antecedent for the cataphoric pronoun, which was consistent with the conclusion drawn from the effects of Gender Match and its interaction with Constraint.

Further evidence supporting Chinese EFL learners’ late application of Binding Principle B was also found in late eye-tracking data at region 3. In this region, the object elicited more regressions in no-constraint than in constraint conditions. In no-constraint conditions, both the subject and object could be the antecedent of the cataphoric pronoun in terms of their syntactic position. In constraint conditions, however, only the object was licensed to co-refer with the cataphoric pronoun. If Chinese EFL learners abided by Binding Principle B in the late processing stage, the matrix subject would not interfere with the late integration stage during cataphora resolution in constraint conditions, but would exert its influence in no-constraint conditions. As a result, more regressive eye-movements from the object would be made in no-constraint than in constraint conditions which was just the pattern we observed in regression path durations at region 3. Besides, this effect of Constraint kept constant at region 4 as indicated by longer regression path durations and total reading times in no-constraint than in constraint conditions. In spite of Chinese EFL learners’ late application of the binding constraint, however, the gender feature of the syntactically-illicit name (i.e., matrix subject in constraint conditions) could still exert its influence on the integration stage in cataphora resolution, which can be seen from the longer total reading times in constraint, gender-match conditions than in constraint, gender-mismatch conditions at region 3.

It is worth-noting that a significant Constraint by Gender Match interaction was also observed in first fixation durations at region 1 (matrix subject name). We did not take this interaction as evidence that Principle B was early applied by Chinese EFL learners to constrain the cataphora resolution process because this interaction was only found in first fixation durations at region 1 and was absent in other early eye-movement measures in this region and its spillover region (region 2). Considering the experimental materials used in our experiment were quite different from what were used in previous cataphora resolution studies, the interpretation of this seemingly odd interaction should start from the structure of our own materials. It might be related to the process in which matrix subject is first assigned as the subject of the adjunct clause. This may also explain why the effect of Gender Match manifested itself in the form of longer reading times in gender-mismatch than in gender-match conditions. A detailed explanation is given in the General Discussion part.

The experimental data also show that learners’ proficiency did not influence their processing pattern in cataphora resolution. Except for the overall longer reading times observed in LG learners, LG and HG learners behaved remarkably alike in terms of the time-course during which Binding Principle B was applied to constrain their cataphora resolution process, suggesting that learners’ proficiency did not affect when the syntactic constraint was applied in cataphora resolution. However, it seems that LG learners are less skilled in applying Binding Principle B than HG learners during late stages of cataphora resolution, as indexed by the Constraint and Group interactions observed for total reading times at both region 1 and region 3. Moreover, the Constraint and Group interaction observed for first pass reading times at region 2 seems to suggest that LG learners have more difficulty in retrieving the gender feature of the cataphoric pronoun when they started the cataphora resolution process just after finishing the interpretation of PRO. This is supposed to arise from the fact that the LG learners in our Experiment have lower working memory than HG learners. L2 learners’ working memory has been found to positively correlate with their proficiency (van den Noort et al., 2006).

In brief, Binding Principle B constrains Chinese EFL learners’ cataphora resolution process, but just works at late stages of cataphora resolution. As a whole, the time-course of learners’ application of Binding Principle B in English cataphora processing is not modulated by their proficiency.

## 4. General discussion

The two eye-tracking studies aim to investigate how Chinese EFL learners with different English proficiency resolve English cataphoric pronouns and whether their cataphora resolution process is restricted by the syntactic constraint Binding Principle B. Experiment 1 shows that Chinese EFL learners initiate an active search in processing the cataphoric pronouns, yet along a processing path distinct from English natives, and HG learners initiate the active searching process earlier than LG learners. Experiment 2 shows that Binding Principle B constrains Chinese EFL learners’ cataphora resolution process, but works only at later stages of cataphora resolution, unlike the early application of Binding Principle B by English natives in cataphora resolution.

**Table 1:** A comparison of results between English natives and Chinese EFL learners in Experiment 1.

Note: M1: Match M2: Mismatch		English natives	Chinese EFL learners	
			HG	LG
Region 1	FFD	–	–	–
	FPD	–	M1 > M2	–
	RPD	–	M1 > M2	–
	TRT	M1 < M2	M1 > M2	–
Region 2	FFD	–	–	–
	FPD	–	M1 > M2	–
	RPD	–	–	–
	TRT	M1 < M2	M1 > M2	M1 > M2

**Table 2:** A comparison of results between English natives and Chinese EFL learners in Experiment 2.

Note: C1: Constraint C2: No-constraint M1: Match M2: Mismatch		English natives	Chinese EFL learners	
			HG	LG
Region 1	FFD	–	C2M2 > C2M1	C1M2 > C1M1
			–	C2M2 > C2M1
			C1M1 > C2M1	C1M1 > C2M1
	FPD	–	–	–
	RPD	–	–	–
	TRT	C2M2 > C2M1	–	C2M1 > C1M1
		C2M1 > C1M1		C2M2 > C1M2
		C2M2 > C1M2		
Region 2	FFD	–	C1M1 > C2M1	C1M1 > C2M1
			C1M2 > C2M2	C1M2 > C2M2
			C1M2 > C1M1	C1M2 > C1M1
			C2M2 > C2M1	C2M2 > C2M1
	FPD	–	C1M2 > C1M1	C1M2 > C1M1
			C2M2 > C2M1	C2M2 > C2M1
				C1M1 > C2M1
				C1M2 > C2M2
	RPD	–	C2M2 > C1M2	C2M2 > C1M2
	TRT	C2M2 > C2M1	C2M1 > C1M1	C2M1 > C1M1
			C2M2 > C1M2	C2M2 > C1M2
			C1M1 > C1M2	
			C2M1 > C2M2	

### 4.1. Active search mechanism adopted by Chinese EFL learners in cataphora resolution

Just as English natives, Chinese EFL learners also trigger an active search for the antecedent upon encountering an English cataphoric pronoun. However, the specific searching process is quite different from that of English native speakers. Reading times of English natives are slowed down by a gender-mismatching name at the matrix subject position while reading times of Chinese EFL learners are slowed down by a gender-matching name at the same position. This unexpected gender-match effect contradicts the gender-mismatch effect that has been consistently found in previous studies except for Bertenshaw (2009). Bertenshaw (2009) ascribed the gender-match effect observed in Japanese EFL learners to the influence of learners’ first language in which disjointed coreference between the cataphoric pronoun and the first name was preferred in the context of experimental sentences. However, this explanation cannot account for the gender-match effect in our study for two reasons. The first reason is that experimental sentences in Bertenshaw’s study differed from what we used. In Bertenshaw’s (2009) study, a lead-in sentence containing one male name and one female name preceded the critical sentence containing the cataphoric pronoun, which made the pronoun not strictly cataphoric since it can access interpretation from the male or female name based on its gender co-indexation. Therefore, the existence of the lead-in sentence might be the real reason that Japanese EFL learners’ first language had an impact on their interpretation of the cataphoric pronoun. The second reason is that Chinese EFL learners do not possess prior knowledge regarding the resolution of cataphoric pronouns, for there is no intra-sentential cataphora in Chinese (Wang, 1994; Wang, 2000, 2006; Zhao and Shao, 2002). Then how to account for the gender-match effect in our study? One possibility is that Chinese EFL learners are affected by their awareness of first language in the option of processing strategies in cataphora resolution. The transfer of L1 processing strategies in L2 sentence processing has been confirmed by a number of studies (e.g., Gass, 1987; Harrington, 1987; Kilborn and Cooreman, 1987; Kilborn, 1989; Hernandez et al., 1994; Su, 2001). As Chinese is a semantics-driven language (Xu, 1999), Chinese native speakers are more concerned with semantic congruity than syntactic congruity when processing sentences. This processing preference might be transferred to their L2 sentence processing, which makes them less error-tolerant in semantic plausibility than English natives upon encountering a gender-mismatching potential antecedent in cataphora resolution. As mentioned above, before the gender feature of the matrix subject name becomes available, a link between the cataphoric pronoun and the name has already been established by both English native speakers and Chinese EFL learners. When the subject name mismatches the cataphoric pronoun in gender, Chinese EFL learners can quickly revoke the previously established coreference relation and stop taking the subject name as a potential antecedent. By contrast, when the information of that name matches the cataphoric pronoun in gender, they retain the link and engage in a deep processing of the subject name. As a result, more reading time was spent on a gender-matching subject name compared to a gender-mismatching name. However, a gender-mismatching subject name cannot make English native speakers abandon the established coreference relation in a short time as they are more error-tolerant than Chinese EFL learners in English sentence processing. Consequently, whether the subject name matches or mismatches the cataphoric pronoun in gender, English native speakers keep engaged in a deep processing of the name, yielding greater processing difficulty at the gender-mismatching relative to the gender-matching name region. From a cognitive perspective, the L1 transfer found in our study is conceptual transfer whose basic idea is that a person’s comprehension and production of one language is affected by the concepts and conceptualization patterns he acquires in another language (Jarvis, 2007, 2011). Experientialism argues that although people of different nations share the ability to conceptualize their experience, they have developed different conceptual systems due to differences in their specific life experiences, environments, and cultures (Lakoff, 1987). Therefore, Chinese natives differ from English natives in terms of their specific conceptual system and conceptual patterns, as revealed by their language typology (Chinese, a semantics-first language contrasts English, a syntax-first language). As a result, Chinese EFL learners’ processing of English sentences in our study is subject to the influence of their native language.

At the object name region (region 3), the regression path durations and total reading times were significantly longer in gender-match conditions than in gender-mismatch conditions. This can be explained by the Competition Model (McDonald and MacWhinney, 1995). According to the Model, processing should be much more difficult when there are two potential antecedents than when there is only one for the anaphoric pronoun, because the two potential antecedents might compete for the final interpretation of the anaphoric pronoun whereas there is no competition involved when only one potential antecedent is available. In our Experiment 1, the object name in gender-match conditions serves as another potential antecedent for the cataphoric pronoun, competing with the subject name for being chosen as the final interpretation of the cataphoric pronoun during later stages of cataphora resolution. In contrast, the object name in gender-mismatch conditions is the only appropriate antecedent for the cataphoric pronoun and no competition will arise between the object name and the subject name, for the gender feature of the subject name clashes with that of the cataphoric pronoun. Evidently, Competition Model can not only be used to reveal the processing mechanism behind ambiguous forwards anaphora resolution, but also holds explanatory power in ambiguous cataphora resolution as in our study.

Apart from the two above-mentioned findings, Experiment 1 also demonstrated a modulating effect of learners’ proficiency on the time-course of their active searching process. Specifically, LG learners delayed their active searching process relative to HG learners. Following the above-mentioned L1 transfer account, LG learners should be more affected by their L1, thereby showing earlier and stronger GME effect. However, what we found was just the opposite. HG learners exhibited earlier and stronger GME effect than LG learners, which means there was another force playing a greater role. This dominating force stems from learners’ employment of active search mechanism in cataphora resolution. As we have discussed in the preceding text, readers’ employment of an active search mechanism implies two steps: the first step is to predictively establish the coreference relationship between the cataphoric pronoun and the matrix subject position, and the second step is to access the gender feature of the matrix subject to confirm their analysis. Accordingly, two reasons might help to explain our results. One is related to LG learners’ reduced predictive ability compared to HG learners in L2 sentence comprehension, which has been demonstrated by several studies (e.g., Kaan, 2014; Peters et al., 2015). As a result of LG learners’ reduced predictive ability, the predictive process of linking the cataphoric pronoun to the matrix subject position (region 1) might be slower and more cognitive-demanding for LG learners than HG learners. The other reason for our results might be that LG learners are slower and less efficient than HG learners in accessing the gender features of the matrix subject. According to the revised hierarchical model (RHM), L2 and L1 words in the bilingual mental lexicon share one conceptual system while their corresponding forms are represented separately (Kroll and Stewart, 1994). Several scholars (Chen, 1990; Dufour and Kroll, 1995; Cheung and Chen, 1998) interpreted the RHM from a developmental perspective and held that L2 learners with lower proficiency access L2 word meaning through L1 lexicon while learners with higher proficiency access L2 word meaning directly *via* the shared conceptual system, the claim being confirmed by several studies (e.g., Jared and Kroll, 2001; Kroll et al., 2006, 2010). Therefore, it is rational to assume that LG learners in our study are slower than HG learners in accessing the gender information of the matrix subject. In a word, LG learner’s reduced predictive ability and slower access to the gender information of the matrix subject together contribute to their relatively delayed employment of the active search mechanism in cataphora resolution.

### 4.2. Late application of principle B by Chinese EFL learners in cataphora resolution

Chinese EFL learners’ cataphora resolution process was constrained by Principle B. However, unlike English native’s early application of Principle B, Chinese EFL learners, irrespective of their proficiency levels, resorted to Principle B to resolve cataphora at a later stage. Before further exploring the underlying reasons for the different processing patterns between Chinese EFL learners and English native speakers, we first take a look at the seemingly odd Gender Match and Constraint interaction observed in first fixation durations at region 1. As we have mentioned in 3.6, the interpretation of this interaction should be based on the structure of the experimental materials used. For better illustration, the exemplar sentences in Experiment 2 are repeated here as (7).

(7) a. constraint, gender-match/mismatch

After PRO buying her/him an iced coffee, Linda generously offered Elsa/Victor the best seat at the meeting table.

b. no-constraint, gender-match/mismatch.

After PRO buying her/his colleague an iced coffee, Linda generously offered Elsa/Victor the best seat at the meeting table.

The experimental sentences involve two types of cataphoric relations, one of which is between PRO and the matrix subject and the other is between the cataphoric pronoun and its antecedent. Using the same materials, Kush and Dillon (2021) demonstrated that English native speakers could process the two types of cataphoric relations simultaneously by incrementally integrating syntactic and pragmatic information. However, our experimental data suggest that Chinese EFL learners could only process the two cataphoric relations one by one. Though a couple of studies claim that cataphoric pronouns are not used in Chinese (Wang, 1994; Wang, 2000, 2006; Zhao and Shao, 2002), some scholars take a different view in this issue (Xu and He, 2007; Gao, 2010; Yu, 2011). For instance, Gao (2010) held that cataphoric pronouns existed in Chinese in an invisible and inaudible way, i.e., zero cataphoric pronouns are present in Chinese. Gao (2010) conducted a corpus-based contrastive study of cataphora in Chinese and English, and found that cataphora in Chinese manifested itself only in the form of zero pronouns while cataphora in English existed in the form of both zero and overt pronouns. At the same time, zero cataphoric pronouns in Chinese and English are used in a similar way in which the invisible pronoun appears in a preposed modifier and the antecedent appears in the matrix subject position or as a modifier of the matrix subject. The PRO in our experimental sentence is just the kind of zero cataphoric pronouns Gao (2010) has identified. Since this kind of zero cataphoric pronouns exists in both Chinese and English and is used in a similar way across two languages, Chinese EFL learners might prioritize the processing of PRO (i.e., zero cataphoric pronoun) over the processing of the overt cataphoric pronoun which is rarely used in Chinese. Following this assumption, we can well explain the seemingly odd Constraint and Gender Match interaction observed in first fixation durations at region 1. Specifically, during the early processing of region 1, Chinese EFL learners only completed the interpretation of PRO, assigning it to the matrix subject (region 1) while the resolution of the cataphoric pronoun had not yet started at this time. After the matrix subject was interpreted as the subject of the subordinate clause, the meaning of the subordinate clause in no-constraint, gender-match conditions was accessed more easily than the meaning of the adjunct clause in no-constraint, gender-mismatch conditions due to the gender-incongruency between the matrix subject and the cataphoric pronoun, thereby resulting in significantly longer first fixations in no-constraint, gender-mismatch conditions than in no-constraint, gender-match conditions. At the same time, the process of assigning the matrix subject name to PRO made the gender feature of the matrix subject available to the parser before the resolution of cataphoric pronouns started, consequently leading to longer reading times being spent on a gender-mismatching subject name during cataphora resolution, contrastive to the GME effect observed in Experiment 1 where coreference between the cataphoric pronoun and the matrix subject was already established even before the gender information of the subject name becomes available to the parser. In addition to a Constraint and Gender Match interaction, there came up a significant Gender Match and Group interaction for first fixation durations at region 1. Further analysis on this interaction indicated that LG learners were more easily disrupted than HG learners by the gender feature of the cataphoric pronoun stored in their working memory when assigning the matrix subject to PRO.

Chinese EFL learners behave themselves differently from English natives in using Binding Principle B from the perspective of time course regarding cataphora resolution. Concerning the time-course of syntactic constraints in cataphora resolution, there are two major hypotheses. One is the early filter hypothesis, claiming that syntactic constraints play an early role in the parser’s active searching process so that only positions licensed by the grammatical constraints can be predictively considered as holding potential antecedents. The other one is the delayed filter hypothesis, holding that syntactic constraints are applied at a later processing stage to filter out ungrammatical interpretations, which implies noun phrases at positions not licensed by syntactic constraints are initially considered as potential antecedents. So to speak, the performance of English native speakers in Kush and Dillon (2021)’s study is consistent with the early filter hypothesis while the performance of Chinese EFL learners in our experiment 2 is in line with the delayed filter hypothesis. Why does Principle B function as an early filter in English natives’ cataphora resolution but a delayed filter in Chinese EFL learners’ cataphora resolution? The difference between L1 and L2 sentence processing might be responsible for the discrepancy. According to Shallow Structure Hypothesis (Clahsen and Felser, 2006a,b), L2 learners prioritize semantic and pragmatic cues over syntactic cues to guide their sentence processing, which leads to shallow and less detailed syntactic representations being computed by L2 learners, as compared to native speakers. Given that our experimental sentences involve two types of cataphoric relations, it is rational to assume that Chinese EFL learners have not established the required syntactic representations to correctly resolve the cataphoric pronoun when first encountering the matrix subject. As a result, the EFL learners made the ungrammatical interpretation that is based solely on the gender-match between the cataphoric pronoun and the matrix subject, without taking the syntactic constraint into account.

Previous forwards anaphora resolution studies show that L2 learners tend to delay the application of binding constraints (Felser et al., 2009; Felser and Cunnings, 2012). Targeting at L2 English learners’ online processing of reflexives, both Felser et al. (2009) and Felser and Cunnings (2012) found that L2 learners were initially affected by a discourse-salient but binding-inaccessible antecedent while English native speakers could immediately apply Binding Principle A to resolve reflexives, suggesting that L2 learners’ early processing of reflexives was not restricted by syntactic constraints. In our study, Chinese EFL learners were also found to have delayed their application of the binding constraint in cataphora resolution. Therefore, the delayed application of binding constraints by L2 learners in both forwards anaphora and cataphora resolution adds evidence to the claim that L2 learners have more difficulty than native speakers in establishing nonadjacent syntactic dependencies in real time (Clahsen and Felser, 2006a).

Both Binding Principle B and Binding Principle C are observed by EFL learners in English cataphora resolution. Unlike the delayed application of Binding Principle B by learners in our study, previous cataphora resolution studies demonstrated that native speakers and L2 learners patterned alike in terms of the time-course of their application of Binding Principle C when resolving cataphoric pronouns (Rodríguez, 2008; Bertenshaw, 2009; Drummer and Felser, 2018). This implies that Binding Principle B and Binding Principle C are treated differently by L2 learners. To be more specific, it seems that Binding Principle C was more easily applied than Binding Principle B by L2 learners in their online resolution of cataphoric pronouns. Comparing the materials used in previous L2 cataphora resolution studies and those used in our study, we find that the coreference relations subject to Binding Principle C was more easily detected than those subject to Binding Principle B because the structure of sentences involving Principle C constraint was much simpler than those involving Principle B constraint. On this account, it appears safe to argue that L2 learners’ sensitivity to different binding constraints in cataphora resolution is also dependent on the complexity of sentences involved. Despite L2 learners’ divergent sensitivity to Binding Principle B and Binding Principle C, all L2 learners can follow these two syntactic constraints in the corresponding sentences to exclude ungrammatical antecedents when resolving cataphoric pronouns online.

### 4.3. Chinese EFL learner’s English proficiency and their cataphora processing

Chinese EFL learners’ proficiency did not modulate the time-course of their application of Binding Principle B in cataphora resolution. Specifically, both HG and LG learners delayed their application of the binding constraint when resolving cataphoric pronouns. Nevertheless, the data in Experiment 1 showed that learners’ proficiency affected when they initiated an active search for the antecedent. What is dedicated to the absence of the modulation effect of learners’ proficiency in Experiment 2? We resort to Shallow Structure Hypothesis to explain the absence. The key tenet of Shallow Structure Hypothesis is that L2 sentence processing is fundamentally different from L1 sentence processing and even highly proficient learners cannot achieve native-like level in terms of processing complex syntactic structures like ambiguous relative clauses and filler-gap dependencies while native-like processing of simple syntactic structures like subject-predicate agreement can be attained as the learners’ proficiency increases. As mentioned in the preceding part, the sentences in Experiment 2 involve two types of cataphoric relations and the correct resolution of cataphoric pronouns necessitates the successful representation of these two types of cataphoric relations. In contrast, the sentences in our Experiment 1 are relatively simple and the successful resolution of cataphoric pronouns relies more on whether the name matches the cataphoric pronoun in gender. That is, since the sentences were more complex in Experiment 2 than in Experiment 1, learners’ proficiency modulated their online performance in Experiment 1 but became invisible in Experiment 2.

## 5. Conclusion

We conducted two eye-tracking studies to investigate how Chinese EFL learners with different proficiency resolved cataphoric pronouns and whether their cataphora resolution process was restricted by Binding Principle B. The results revealed that Chinese EFL learners initiated an active search for the antecedent when encountering a cataphoric pronoun, yet along a processing path distinct from English natives. Specifically, Chinese EFL learners predictively linked a cataphoric pronoun to the first potential antecedent, but only a gender-matching antecedent could prompt them to engage in a deep processing of the antecedent, the reason being that concepts and conceptualization patterns Chinese EFL learners acquired in their mother tongue influenced their English sentence processing. In addition, learners’ proficiency modulated the time point at which they initiated an active searching process, with HG learners earlier than LG learners. Moreover, our results demonstrated that Chinese EFL learners’ cataphora resolution process was restricted by Binding Principle B, but the application of Binding Principle B was delayed in both HG and LG learners compared with English natives, suggesting that Chinese EFL learners were not so much sensitive to syntactic constraints as English natives in cataphora resolution.

To conclude, this study demonstrated how Chinese EFL learners resolve cataphoric pronouns. By extending cataphora resolution studies to a group of English learners with a distinct L1 (i.e., Chinese) in typology, we provide more insights into L2 sentence processing and adds more evidence to the Shallow Structure Hypothesis. Subsequent studies are expected to explore how English learners with various L1 backgrounds resolve cataphoric pronouns, as well as whether other factors such as learners’ working memory influence their online performance on resolving cataphoric pronouns, so as to provide a real panorama for the L2 cataphora resolution mechanism.

## Data availability statement

The original contributions presented in the study are included in the article/Supplementary material, further inquiries can be directed to the corresponding authors.

## Ethics statement

The studies involving human participants were reviewed and approved by Ethics Committee of Qufu Normal University. The patients/participants provided their written informed consent to participate in this study.

## Author contributions

TW, MG, and YW conceived the study. MG, YW, and MZ performed the experiments. MG collated and analyzed the date. TW and MG drafted the first manuscript, which was revised by TZ and YY. All authors contributed to the article and approved the submitted version.

## Funding

This work was supported by the National Social Science Fund of China under grant 21BYY103.

## Conflict of interest

The authors declare that the research was conducted in the absence of any commercial or financial relationships that could be construed as a potential conflict of interest.

## Publisher’s note

All claims expressed in this article are solely those of the authors and do not necessarily represent those of their affiliated organizations, or those of the publisher, the editors and the reviewers. Any product that may be evaluated in this article, or claim that may be made by its manufacturer, is not guaranteed or endorsed by the publisher.

## Footnotes

^1^early eye-tracking data at region 3 and region 4 were excluded from further discussion since only the late processing of those regions was related to our research questions.
